# Implementation of a clinical research “One Signature Initiative” at a large academic medical center

**DOI:** 10.1017/cts.2024.667

**Published:** 2024-11-29

**Authors:** Elizabeth Elin Robison, Trina Lynn Moy, Katelyn Leanna Trigg

**Affiliations:** UC Davis Health, Sacramento, CA, USA

**Keywords:** Training, regulatory, clinical trial management, delegation of authority, quality assurance

## Abstract

A clinical research team’s goal is to support the implementation, conduct, and monitoring of research studies and corresponding protocols. There is a need to ensure that study teams have adequate resources and regulatory support to successfully adhere to regulations and good clinical research practices. Our team, the UC Davis Division of Infectious Diseases Research Unit (IDRU), sought to establish a One Signature Initiative program for all studies and protocols supported by the IDRU. The One Signature Initiative designates one point of contact from each ancillary team or department to sign delegation and training logs, who in turn is delegated to train their team. The goals of the One Signature Initiative were, and are still, to reduce task redundancy, lessen regulatory burden on research teams, and minimize audit findings. Since the implementation of the One Signature Initiative in 2023, acceptance has been favorable, and we have expanded its footprint by incorporating it into our standard operating procedures. This article discusses our experiences, and ancillary departments’ experiences, with the One Signature Initiative. Our experience is an example of how a One Signature Initiative can be developed that is efficient, effective, and well-accepted by clinical research stakeholders.

## Introduction

Conducting clinical trials and studies involves numerous stakeholders outside the coordinating team. Many large academic medical centers (AMCs) have a centralized process for clinical trial management. However, not all AMCs have the infrastructure to centralize clinical research management. A lack of organizational structure to support research within the confines of the academic teaching hospital and its relationship with its university (or other academic counterpart) can lead to inefficiencies [1].

At our institution, clinical research is not centralized, which means that each individual department (and even divisions within departments, e.g., Infectious Diseases within Internal Medicine) operates independently. While this can allow for more autonomy to conduct research, having a decentralized structure can also be limited to smaller departments or divisions trying to develop or grow a clinical research portfolio, as well as hamper the ability to scale up initiatives and cross-departmental or – disciplinary focus.

Oftentimes the coordinating team is asked to furnish proof of training and experience of ancillary staff. This places a significant burden on the coordinating team to obtain multiple signatures on delegation and training documents not only at study startup, but with each protocol amendment. This is not feasible and sometimes physically impossible.

Gaps and inefficiencies become especially apparent when implementing multidisciplinary studies [2]. For instance, the Clinical and Translational Science Award program funded by the National Institutes of Health provides support for major research institutions across the country, including UC Davis. The program encourages team science and innovative projects requiring expertise from multiple fields and is a boon to scientific discovery. However, team science increases the number of project members dramatically and thus exacerbates the regulatory burden on the coordinating team.

## The problem

AMCs employ thousands of staff and clinicians; it is not feasible for coordinating teams to require additional research duties of ancillary staff who are performing assessments and procedures within their typical clinical scope [3].

Consider a sponsor requiring that a bedside nurse be delegated, trained, and have a Good Clinical Practice (GCP) certificate in place before administering a drug that is otherwise within their scope of practice to administer. It is not uncommon for nurses who were assigned to a patient on Monday to not see that patient again for the entirety of the patient’s stay. If the drug is to be administered twice daily for 2 weeks and nurses rotate every 12 hours, it is possible for up to 28 nurses to then be “required” to be added to the study.

## Implementation of the Initiative

Our internal quality assurance team and research management developed a standardized process for obtaining signatures from ancillary staff, here referred to as the One Signature Initiative, or simply the Initiative.

First, our team determined workflows to decide which studies may utilize the process. This flow consists of questions such as:

- Does the sponsor require ancillary staff to be protocol-trained?
- Does the sponsor require ancillary staff to complete GCP training?
- Does the sponsor require ancillary staff to be delegated protocol-defined duties?

Due to the Initiative’s innovative nature, communication was an instrumental component of its success both with internal teams and across sponsors.

Second, the Initiative asks the coordinating team to obtain signatures from a single point of contact (POC) within a designated team or department. Examples from our institution are a manager of Respiratory Care, an assigned pharmacist in the Investigational Drug Services (IDS) pharmacy, or a nurse manager from a hospital unit. The POC is a member of the study team, and the coordinating team must train, delegate, and obtain current curriculum vitae (CVs) and applicable licensure from them.

Third, this POC is delegated to act as the lead individual in an ancillary department and is responsible for training their team and maintaining a record of their team’s training. It is worth clarifying here that the POC is delegated to track their team’s compliance with the study protocol, but ultimately it is the Principal Investigator’s responsibility.

## Amendments

The One Signature Initiative has also addressed the concerns and inefficiencies surrounding retraining and capturing additional signatures when there are protocol amendments [4,5]. The One Signature Initiative incorporates language that retraining of the POC only occurs in instances where there are updates to their scope or specific changes to the procedures or assessments they’ll be conducting.

For example, if there is a protocol amendment that adds or removes procedures to visits that do not impact the ancillary department, it would not trigger a need for retraining. However, if a study adds a cohort where study drug will be given, it is likely that the investigational pharmacy would require an amendment to their process and thus a retraining on that new cohort.

Instances of retraining are uncommon and highly variable in study design and primary endpoints. Our team has not seen this to be a limiting factor in either uptake or support of the One Signature Initiative by the coordinating team.

## Uptake

Post-implementation, the study team began soliciting feedback from internal departments on its uptake and use. This feedback was collected by One Signature Initiative authors KLT and EER via informal email interview. Interestingly, the uptake among most groups (five of the six) was positive (see Table 1).

**Table 1.** Ancillary dept comments on the one signature initiative

Ancillary Dept	Amount of time the depts have used the Initiative	Pro	Con	Mitigation
1. Investigational Drug Services (IDS)	1 year	Fully supportive, reduces the burden of paper logs and they prefer to use their electronic system (nCoup) to document trainings.	Concerns about the time required to initially setup the training log in nCoup. Concerns that the sponsor would not grant individuals access to IRT if not listed on Delegation of Authority log.	The Coordinating team agreed to inform them of the use of the Initiative during study Start-Up. The One Signature Initiative was amended to specify that all members of the ancillary dept are expected to receive system access as appropriate to conduct the study.
2. Alpha Stem Cell Clinic	9 months	No comment.	Concerns about the time required to setup and maintain the training logs.	The Coordinating team agreed to provide this team with template logs to reduce the required effort.
3. Pathology and Laboratory Medicine	1 year	Fully supportive.	No comment.	
4. CTSC Clinical Research Center	1 year	Fully supportive, stated success with utilizing this setup with other departments.	No comment.	
5. Floor Nurses	3 months	Fully supportive.	No comment.	
6. Respiratory Therapists	3 months	Fully supportive.	No comment.	

There was one department, however, that was initially concerned about regulatory compliance. Given the considerable clinical demand on their time, they were apprehensive about the perceived additional effort required of them to maintain training logs. To mitigate this concern, the coordinating team supported them by providing template logs which reduced their additional effort.

The IDS team was completely supportive of the Initiative. They asked the coordinating team to inform them of the Initiative’s use before study implementation. By notifying them ahead of the Site Initiation Visit, this allowed IDS to setup the training log and document their teams’ training before sponsor representatives were on-site. They also requested that all pharmacists be granted access to Interactive Response Technology, which the One Signature Initiative was amended to support.

UC Davis Institutional Review Board Administration leadership reviewed the One Signature Initiative and had suggestions on defining the Initiative for clarity among stakeholders as well as describing the initiative more clearly within the purpose section of the Initiative. They also suggested including the Initiative’s goal in its application among internal ancillary departments and groups. They felt the Initiative is meaningful to define roles and responsibilities when research requires collaboration between departments and administratively helpful to departments and study teams. UC Davis Compliance does not typically provide feedback on Department-specific policies and procedures; however, we did solicit their feedback on this SOP, but they remained silent on providing any comments or feedback stating it is outside of their scope and no portion of the policy directly impacts compliance.

Additionally, the One Signature Initiative was provided to our sponsors as our research unit’s standard operating procedure, and sponsors have been amenable to its use. Since implementation, it has been supplied to over 20 different sponsors ranging from small to large manufacturers. To date, we have not had any sponsors unwilling to follow our SOP, however, there have been some instances of study-specific nuances necessitating slight modifications to our policy. For example, one sponsor was hesitant to accept that the Initiative covers all study-specific training, not just protocol training. In this situation, it was necessary to amend the Initiative to specify that ancillary departments may be responsible for maintaining all study-specific training that is applicable to their role (such as the Investigator’s Brochure, Pharmacy manual, Central Laboratory Manual, etc.). Once this was clarified, this sponsor accepted the use of the One Signature Initiative for all study-specific training.

Lastly, within our research unit, this Initiative improved regulatory workflow by streamlining communication with ancillary departments and significantly reduced time spent gathering signatures and regulatory paperwork from ancillary staff.

## Limitations

The One Signature Initiative was created to address operational struggles identified at our large AMC during the COVID-19 pandemic. As such, this policy is aimed at improving clinical research workflow within a large AMC. That is not to say that this could not be applied elsewhere. However, there are a few notable limitations.

The One Signature Initiative must operate within the bounds of rules and regulations set forth by the Code of Federal Regulations, Belmont Report, Nuremburg Code, Declaration of Helsinki, and ICH Harmonized Guideline for GCP. The idea is only as good as its adherence to these important policies.

As such, there are, admittedly, scenarios where the One Signature Initiative cannot be applied based on regulations. For instance, it cannot be applied to:

- Staff obtaining informed consent from participants
- Investigators (Principal Investigators and sub-Investigators)
- Staff administering an intervention outside of their typical clinical scope (e.g., the insertion of a novel medical device via a novel surgical procedure)

For example, fellows and residents could fall under the One Signature Initiative if the only study tasks they perform are within their typical scope, such as a routine physical exam (i.e., they are not listed in the 1572). In fact, if the individual is delegated tasks that are within their typical scope, it may not even be considered research. However, if they are participating in the study as an investigator or making any determinations about adverse events and/or study endpoints, then the One Signature Initiative does not apply.

The POC has significant responsibility under this policy. Should a POC and Principal Investigator disagree on study conduct (for instance, required qualifications, training standards, protocol interpretation), the ultimate decision and responsibility lies with the Principal Investigator, as with all clinical research. The One Signature Initiative does not change this. We encourage study teams to have transparent conversations with ancillary departments about the study protocol’s needs and the ancillary department’s typical scope of work, to determine if their department’s role in the study can fall under the One Signature Initiative.

In the absence of a broad endorsement from a national regulatory authority, the generalizability of the One Signature Initiative can only be determined by implementing a similar pilot program at other research centers. The unique combination of institutional infrastructure, regulatory policies, study stakeholders, and specific study needs will determine whether the Initiative is well-received. What proved effective in our setting may not be directly transferrable to other institutions. More research is needed to determine whether this Initiative could be applied in settings outside of AMCs, such as Veterans Affairs hospitals, private research clinics, and decentralized sites. However, these research locations have the same challenges as AMCs when it comes to being understaffed and overburdened, so the Initiative has the potential to be highly valuable.

At the end of the day, we must use our ethical judgment; consult with trusted peers, sponsors, and regulatory authorities; and remain focused on protecting the rights, safety, and welfare of study subjects while advancing scientific research in a manner that is sustainable for research staff and the broader research community.

## Conclusion

The One Signature Initiative improved workflow within our department and between ancillary departments. The Initiative reduces regulatory burden and allows the coordinating team to collaborate more efficiently with ancillary departments when their role in the study is within their typical scope of work. Our research unit can provide this SOP to assist other research units in developing a sustainable clinical research program. More research is needed to determine the applicability of the Initiative at other types of research institutions.

## References

[1] Snyder DC , Brouwer RN , Ennis CL , et al. Retooling institutional support infrastructure for clinical research. Contemp Clin Trials. 2016;48:139–145. doi: 10.1016/j.cct.2016.04.010. 27125563 PMC4889441

[2] Jain RK. Lessons from multidisciplinary translational trials on anti-angiogenic therapy of cancer. Nat Rev Cancer. 2008;8:309–316. doi: 10.1038/nrc2346. 18337733

[3] Bentley JD , Chusid J , D’Antuono GR , Kelly JV , Tower DB. Faculty practice plans: the organization and characteristics of academic medical practice. Acad Med. 1991;66:433–439. doi: 10.1097/00001888-199108000-00002. 1883424

[4] Getz K , Smith Z , Botto E , Murphy E , Dauchy A. New benchmarks on protocol amendment practices, trends and their impact on clinical trial performance. Ther Innov Regul Sci. 2024;58:539–548. doi: 10.1007/s43441-024-00622-9. 38438658

[5] Getz K. Shining a light on the inefficiencies in amendment implementation. Appl Clin Trials. 2023;32(12): 10–11.

